# Ocular Sporotrichosis: Different Spectrums of Clinical Manifestations and a Review of the Literature

**DOI:** 10.7759/cureus.27186

**Published:** 2022-07-23

**Authors:** Abd Hadi Mohd Rasidin, Wen-Jeat Ang, Raja Norliza Raja Omar, Liza Sharmini Ahmad Tajudin

**Affiliations:** 1 Department of Ophthalmology and Visual Science, School of Medical Sciences, Universiti Sains Malaysia, Kubang Kerian, MYS; 2 Department of Ophthalmology, Hospital Melaka, Melaka, MYS

**Keywords:** sporothrix schenckii species, subcutaneous mycosis, granulomatous conjunctivitis, granulomatous anterior uveitis, ocular sporotrichosis

## Abstract

We hereby describe two cases of patients with ocular sporotrichosis who presented with different spectrums of clinical manifestations. Both patients had an antecedent history of zoonotic and vegetative contact. The first patient presented with acute granulomatous anterior uveitis, and the second patient presented with granulomatous conjunctivitis. Patients received topical antibiotics, steroids, and cycloplegics. Systemic oral antifungals were added until full recovery was achieved. Both cases were treated without any episodes of relapse or recurrence.

## Introduction

Sporotrichosis is a rare type of subcutaneous mycosis caused by *Sporothrix schenckii* [1]. The most common manifestations are subcutaneous lesions [1]. However, ocular manifestations have also been increasingly observed [1]. Ocular presentations often mimic other common ocular diseases, such as conjunctivitis, uveitis, and scleritis [2]. This frequently leads to misdiagnosis and a delay in the initiation of therapeutic treatment. We share two cases of patients with ocular sporotrichosis who presented with different spectrums of clinical manifestations. Both patients had an antecedent history of zoonotic and vegetative contact. The first patient presented to us with acute granulomatous anterior uveitis, which is rare in the spectrum of ocular sporotrichosis, and the second patient presented with granulomatous conjunctivitis. Patients received topical antibiotics, steroids, and cycloplegics. Systemic oral antifungals were added until full recovery was achieved. Both cases were treated without any episodes of relapse or recurrence. Knowledge of the manifestation of ocular sporotrichosis is paramount for prompt diagnosis and treatment to achieve better ocular and visual outcomes.

## Case presentation

Case 1

**Figure 1:**
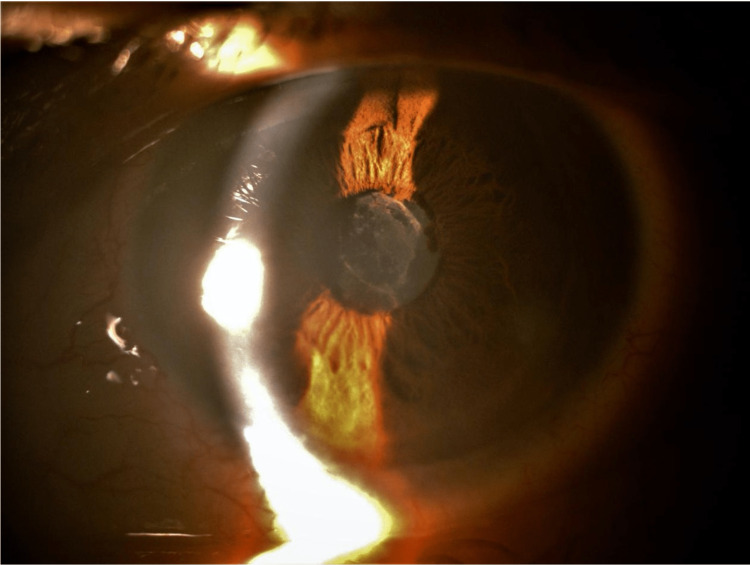
Left eye acute granulomatous anterior uveitis with fibrin formation covering the pupillary aperture.

**Figure 2:**
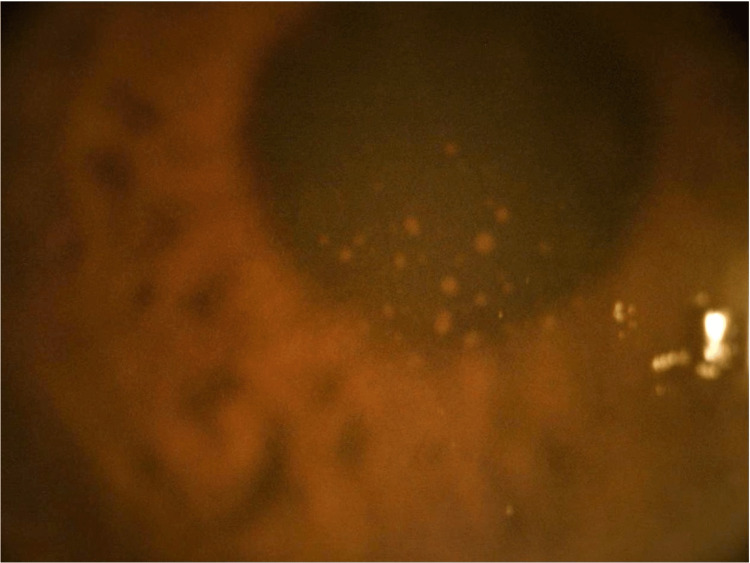
Left eye anterior segment photo of the keratic precipitates forming the Arlt's triangle.

**Figure 3:**
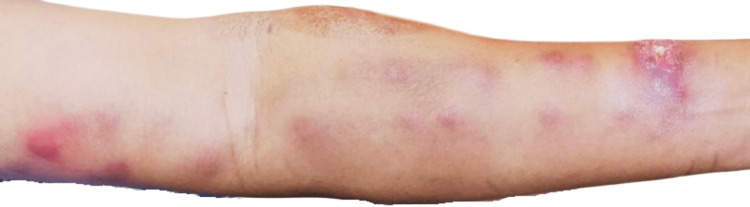
Present of an eroded nodule on the distal part of left forearm extensor representing the primary lesion, followed by multiple erythematous subcutaneous nodular lesion proximal to the primary lesion which arises in a linear ascending pattern.

A 43-year-old male agriculturist with no known comorbidities presented with left eye pain, redness, and photophobia for one week. He stated a history of nonresolving cellulitis over his left forearm after being scratched by a stray cat a month prior to this presentation. The best-corrected vision of the left eye was 6/36 and 6/6 for the right eye. Anterior segment examination showed signs of acute granulomatous anterior uveitis (Figures 1, 2). The fundus view was limited for the left eye and normal for the right eye. B-scan ultrasonography showed no evidence of posterior segment involvement. The intraocular pressure was normal. Dermatological examination revealed an ascending pattern of multiple erythematous subcutaneous nodular lesions over the left forearm (Figure 3). Otherwise, blood investigations, such as full blood count, liver function test, C-reactive protein, and erythrocyte sedimentation rate, were normal, and Venereal Disease Research Laboratory (VDRL) test and Bartonella serology were negative. The Mantoux test reading was 0 mm, and the chest X-ray showed no abnormality.

**Figure 4:**
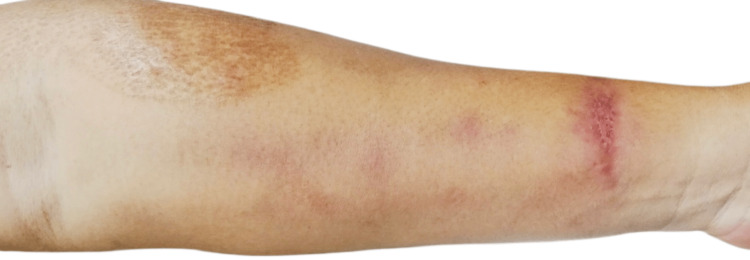
Resolved lymphocutaneous lesion after four months of treatment with oral itraconazole.

A clinical diagnosis of cutaneous sporotrichosis was made, and a daily intake of 200 mg of oral itraconazole was started. Histopathological examination of the skin lesions revealed granulomatous inflammation, which is consistent with sporotrichosis infection. After one month period of oral itraconazole, mycotic skin lesion started to regress and resolved within four months of treatment (Figure 4). Oral itraconazole was subsequently continued for another month for complete clearance of fungal infection. His left eye uveitis was well managed with topical 0.1% dexamethasone eye drops, without any recurrence or relapse.

Case 2

A 60-year-old woman with no comorbidities presented with left eye redness for two weeks. This was associated with nodular conjunctival lesions, which did not respond to treatment with topical steroids and antibiotics. Upon further inquiry, it was found that she had a previous history of a thorn-prick injury across her hands. She denied having any fever, skin infection, or respiratory symptoms.

**Figure 5:**
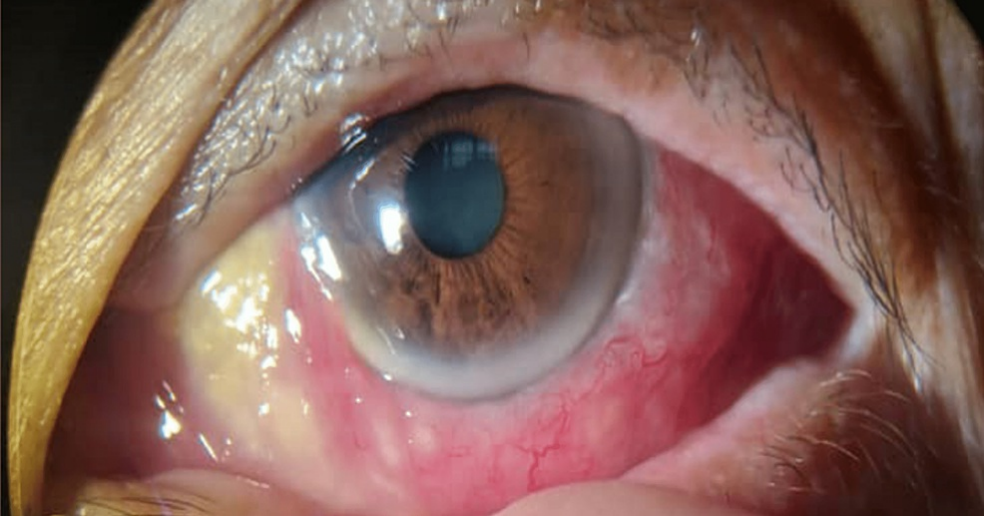
Multiple yellowish conjunctival nodules over nasal side of bulbar conjunctiva of the left eye.

Upon examination, the best-corrected vision was 6/18 for both eyes. Her left eye exhibited generalized conjunctival hyperemia, with multiple yellowish conjunctival nodules on the nasal side of the bulbar conjunctiva (Figure 5). The cornea and anterior chamber were normal. The posterior segment was likewise unremarkable. Systemic examination revealed enlargement of the left preauricular lymph node measuring approximately 1 × 2 cm. The results of routine blood investigations, C-reactive protein, erythrocyte sedimentation rate, rheumatoid factors, and tumor markers were all within normal ranges. The Mantoux test reading was 2 mm, and the chest X-ray showed no abnormality. 

**Figure 6:**
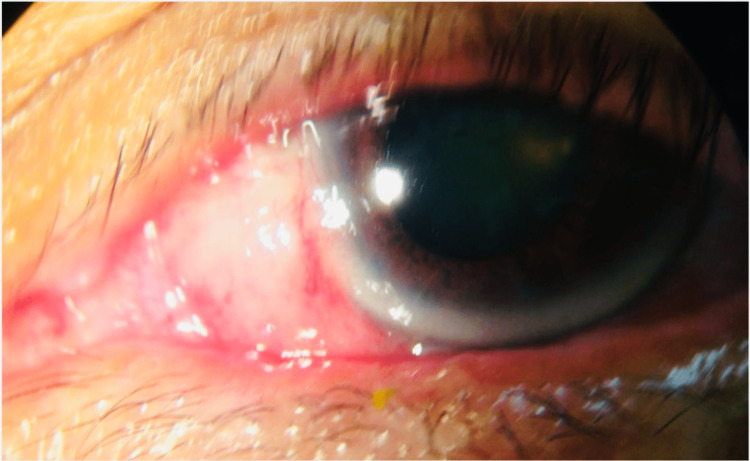
Regression of left eye conjunctival nodules after two weeks of treatment of Akurit 4.

An incisional biopsy of the conjunctival mass was conducted, and histopathological examination revealed an epithelioid granuloma with central caseous necrosis suggestive of mycobacterium infection, which led to the initiation of antituberculosis medication Akurit 4. There was a slight improvement in the conjunctival lesion after two weeks of Akurit 4 (Figure 6). Later, *Sporothrix schenckii* was isolated from a culture of conjunctival tissue. Antituberculosis medication was withheld, and the patient was prescribed 200 mg of oral itraconazole daily for six months. Her condition improved gradually, and the nodular conjunctival lesion completely resolved after five months of treatment with oral antifungal medication. Topical 0.1% fluorometholone was added to control conjunctival inflammation.

## Discussion

The etiological microorganism for sporotrichosis, *Sporothrix schenckii*, commonly penetrates human skin or mucosal layers through traumatic inoculation with organic matter contaminated with the fungus or via zoonotic transmission involving domestic or stray cats [1]. Thus, the prevalence is higher in those working in the agricultural sector. Ocular sporotrichosis involving the ocular adnexa can manifest as granulomatous conjunctivitis, Parinaud oculoglandular syndrome, and dacryocystitis [2]. On the other hand, intraocular forms can present as granulomatous uveitis, retinitis, choroiditis, and endophthalmitis, with reported incidences of blindness due to choroiditis and endophthalmitis [2].

Ocular involvement is generally rare and is almost always induced by trauma. The number of reported cases has been increasing in endemic areas in Brazil and Peru [3]. Eyelid nodules and granulomatous conjunctivitis are the most reported presentations in the eye, while intraocular infection is exceedingly rare and has only been reported in isolated case reports and in a systematic review in the published literature [4]. In Asian countries, the distribution of cases was sporadic, with the largest reported number of cases in China and a limited number of ocular sporotrichosis reports in Japan, Thailand, and Malaysia [5]. 

In Malaysia, sporotrichosis is rare. The latest report by Ahmad-Fauzi et al. described six cases of ocular sporotrichosis that involved the bulbar conjunctiva and the lid [5]. Based on a PubMed literature search, several other case reports of ocular sporotrichosis found that most of the patients who presented with granulomatous conjunctivitis had histories of vegetative or ailing feline contact, where individuals were affected regardless of their immunocompetency status (Table 1). The early commencement of oral itraconazole results in the complete resolution of conjunctival granuloma, whereas late presentation and treatment result in conjunctival fibrosis and the formation of symblepharon. There are a limited number of cases of intraocular sporotrichosis available. One case reported by Cartwright et al. describes a patient diagnosed with pulmonary sarcoidosis who presented with unilateral persistent uveitis. The condition did not respond to topical, oral, and periocular steroids, and the eye condition deteriorated, eventually resulting in endophthalmitis with scleral perforation [6]. *Sporothrix schenckii* was isolated from the vitreous culture, and the patient was treated with intravitreal amphotericin B and topical natamycin [6]. Despite the success in treating fungal infection, due to the late commencement of the antifungal, the affected eye became blind and phthisical.

**Table 1:** Chronologically reported cases of ocular sporotrichosis. *S. schenckii*: *Sporothrix schenckii*; OD: once a day; BD: twice a day; HPE: histopathological examination.

Author	Age/ Gender	Country	Predisposing factor	Clinical presentation	Management	Outcome
Cartwright et al. (1993) [6]	24/Male	USA	History of using a lawn mower	Endophthalmitis, presented as granulomatous uveitis	Vitreous culture: *S. schenckii*, Intravitreal amphotericin B, subconjunctival injection amphotericin B, topical natamycin eye drops hourly	Phthisical eye
Yamagata et al. (2017) [1]	68/Female	Brazil	Gardener	Granulomatous conjunctivitis	Swab culture of skin lesion: *S. schenckii*, oral itraconazole 200 mg OD for nine months	Resolution of conjunctival granuloma with fibrosis of inferior bulbar and tarsal conjunctiva
	46/Female	Brazil	Contact with ailing feline	Granulomatous conjunctivitis	Swab culture of conjunctival lesion: *S. schenckii*, oral itraconazole 200 mg OD for two months, dosage increased to 400 mg OD for another six months due to clinical worsening	Fibrosis and symblepharon over superior bulbar conjunctiva
	14/Male	Brazil	Contact with ailing feline	Granulomatous conjunctivitis	Swab culture of conjunctival lesion: *S. schenckii*, oral itraconazole 200 mg OD	Complete resolution of conjunctival granuloma within 15 days
Ling et al. (2018) [2]	18/Female	Malaysia	Contact with ailing feline	Granulomatous conjunctivitis	Culture of conjunctival biopsy: *S. schenckii*, oral itraconazole 200 mg OD for five months	Complete resolution of conjunctival granuloma
Ahmad-Fauzi et al. (2022) [5]	61/Male	Malaysia	Gardener	Granulomatous conjunctivitis	Initial treatment with intravenous amoxicillin/clavulanic acid and topical ciprofloxacin and ceftazidime therapy, culture of conjunctival swab: *S. schenckii*, change to oral itraconazole 200 mg BD for six months	Complete resolution of conjunctival lesions with symblepharon formation over the inferior conjunctiva
	24/Female	Malaysia	Contact with ailing feline	Granulomatous conjunctivitis	Culture of conjunctival biopsy: *S. schenckii*, oral itraconazole 100 mg OD for two weeks, dosage increased to 200 mg OD for eight weeks due to the development of new conjunctival lesion	Complete resolution of conjunctival granuloma
	39/Female	Malaysia	Contact with ailing feline	Granulomatous conjunctivitis	Culture of conjunctival biopsy: *S. schenckii*, HPE: granulomatous inflammation, oral itraconazole 200 mg BD	Complete resolution of conjunctival granuloma
	17/Female	Malaysia	Contact with ailing feline	Granulomatous lesion over left lower lid	Swab culture of skin lesion: *S. schenckii*, oral Itraconazole 200 mg BD for six months	Complete resolution of granulomatous lesion with residual scarring on the skin
	56/Female	Malaysia	Contact with ailing feline	Granulomatous conjunctivitis	Culture of conjunctival biopsy: *S. schenckii*, HPE: granulomatous inflammation, oral itraconazole 200 mg BD for four months	Complete resolution of conjunctival granuloma
	22/Female	Malaysia	Contact with feline	Granulomatous conjunctivitis	Culture of conjunctival biopsy: *S. schenckii*, HPE: granulomatous inflammation, oral itraconazole 200 mg BD for five months	Complete resolution of conjunctival granuloma
Current study	43/Male	Malaysia	Agriculturist contact with ailing feline	Granulomatous anterior uveitis	Biopsy of a cutaneous lesion, HPE: granulomatous inflammation, oral itraconazole 200 mg OD for five months, topical dexamethasone 0.1% eye drop	Complete remission of anterior uveitis
	60/Female	Malaysia	History of rose thorn-prick injury	Granulomatous conjunctivitis	Empirically treated with Akurit 4 for two weeks, culture of conjunctival biopsy: *S. schenckii*, change to oral itraconazole 200 mg OD for six months, topical fluorometholone 0.1% eye drop	Complete resolution of conjunctival granuloma

In the current reported cases, one had a history of inoculation from contact with a sick stray cat, and another had a history of a rose thorn-prick injury, which were the sources of the traumatic inoculation of *Sporothrix* sp. Nevertheless, each patient presented differently. The approach to the case of ocular sporotrichosis is similar to how the cutaneous form is treated [1]. The diagnosis is made through the collection of conjunctival discharge or lesions, which need to be sent for culturing and histopathological examination [1]. Due to the association with disseminated sporotrichosis, the intraocular form was usually treated with amphotericin B as loading therapy and itraconazole as maintenance, except for cases where endophthalmitis occurs, which requires intravitreal/intravenous amphotericin B [1]. Sporotrichosis affecting the ocular adnexa should be treated with itraconazole of the same dose used for the cutaneous form, i.e., 100-200 mg per day, until complete resolution of the lesions is achieved, typically in three to six months [1].

In Case 1, because the intraocular involvement was limited to the anterior segment, the patient was treated with 200 mg of oral itraconazole daily for five months until the cutaneous lesion of sporotrichosis resolved to nullify the etiological *Sporothrix* sp. This allowed for adequate control of the anterior chamber inflammation of the left eye using topical dexamethasone. In Case 2, the patient was preemptively put on Akurit 4 due to the increasing prevalence of tuberculosis in the country. The clinical and histopathological examinations of the conjunctival mass biopsy also showed granulomatous changes with central caseous necrosis which strongly suggested the presence of *Mycobacterium tuberculosis*. However, the fungal culture that was obtained much later identified *Sporothrix schenckii*. Despite this, a reduction of the conjunctival granuloma lesion was still observed, which suggests the possibility of a cross-reaction between antituberculosis drugs and fungal growth. In vitro studies on the effect of antituberculosis drugs on *Coccidioides posadasii*, which is a dimorphic fungus similar to *Sporothrix* sp., showed that the first-line antituberculosis drugs, alone or in combinations, interfered with the vegetative growth of dimorphic fungus strains [7].

As for Case 2, based on the fungal culture obtained, Akurit 4 was withheld after two weeks of initiation and changed to 200 mg of oral itraconazole 200 mg daily, which led to a significant reduction of the conjunctival granuloma lesion. The delay in proper definite diagnosis and management in both cases may have led to severe and sight-threatening complications, such as fungal endophthalmitis and panophthalmitis. 

## Conclusions

Clinicians should be aware of the rare ocular manifestation of this zoonotic infection transmitted by infected felines and traumatic inoculation from organic matter, as it is reversible with a timely diagnosis and the initiation of antifungal therapy. Further studies may need to be conducted to evaluate the in vitro and in vivo effects of antituberculosis drugs on *Sporothrix schenckii*.
